# Prevalence of echocardiographic left atrial enlargement among hypertensive Nigerian subjects

**DOI:** 10.4314/ahs.v22i2.29

**Published:** 2022-06

**Authors:** Adeseye A Akintunde

**Affiliations:** 1 Department of Medicine, LadokeAkintola University of Technology & LAUTECH Teaching Hospital, Ogbomoso, Nigeria; 2 Goshen Heart Clinic, Osogbo, Nigeria

**Keywords:** Left atrial enlargement, hypertension, prevalence, Nigeria

## Abstract

**Background:**

Left atrial enlargement (LAE) predispose to arrhythmias, atrial thrombogenesis and cardioembolic stroke. Whether LAE constitute a major risk among African hypertensive subjects is not well described. This study was aimed to describe the epidemiologic pattern of LAE among hypertensive subjects and determine their correlates.

**Methods:**

Clinical and demographic details of 414 hypertensive subjects used were taken. Echocardiography was done. LAE was defined as Left atrial dimension (LAD)>3.7 cm. Statistical analysis was done using SPSS 17.0.

**Result:**

414 subjects including 258 (62.3%) males were recruited. The mean age was 56.8±16.8 years. LAE was present in 57.73% of hypertensive subjects. Those with LAE were likely to be older (58.23±14.5 vs. 54.8 ±19.7 years, p<0.05), had a higher waist circumference (88.1 ±26.8 ±75.8 ±28.4 cm, p<0.05), left ventricular mass index (79.2 ± 12.4 vs. 48.7 ±15.5g/m2.7, p<0.05) and a higher frequency of left ventricular hypertrophy (LVH) (65.3% vs. 40.0 %, p<0.05) respectively than those without LAE. LAD was significantly higher among those with LVH than those without LVH (41.4 ±8.4 vs. 35.6 ±5.9 mm respectively, p<0.05).

**Conclusion:**

LAE is common among Nigerian hypertensive subjects. Age, waist circumference, left ventricular wall dimension and mass index are the important correlates of LAE in hypertensive Nigerians.

## Introduction

The left atrium is a conduit between the four pulmonary veins and the left ventricle allowing passive inflow of blood to the left ventricle (LV) during early diastole. The atrial systole accounts for about 20–30% of the LV inflow occurring in late diastole.^1^ Due to its structural nature, left atrial abnormalities may result in atrial fibrillation/flutter with increased risk for cardioembolic (ischaemic) stroke.^2^–^4^ The left atrial appendage is a low velocity apartment with increased predisposition to thromboembolism.^5^ Left atrial enlargement/ abnormalities have been shown to be predictors of cardiovascular (CV) outcome in many studies in different population.^3^,^6^–^8^ There is also a correlation between left atrial size and its functions.^3^,^7^ Current evidences suggest that echocardiographically determined LA size may become an important clinical risk marker in preclinical CV disease and should be assessed as part of routine comprehensive echocardiographic evaluation.^6^,^8^ Despite these evidences, left atrial dimension, function and associated correlates are rarely used in risk stratification among African hypertensives. Some authors have demonstrated that hypertension is associated with left ventricular diastolic dysfunction which is an indirect index of left atrial abnormality among Africans.^9^,^10^ Few others have shown the involvement of the left atrium in hypertension among Blacks.^10^–^11^ Data are scarce on the clinical correlates of left atrial enlargement/abnormalities among treated hypertensive subjects in Nigeria. This study was aimed at determining the frequency of LAE among Nigerian hypertensive subjects and its associated clinical correlates.

## Materials and methods

This was a retrospective review of echocardiography done at the LadokeAkintola University of Technology Teaching Hospitals, Osogbo&Ogbomoso and Goshen Heart Clinic, Osogbo, all in South West Nigeria. Echocardiography was done as part of the cardiovascular risk assessment of hypertensive subjects. Echocardiography was done according to standardized protocols as recommended by the American Society of Echocardiography. 12 Two dimensional, Colour Doppler, pulse wave Doppler and M-mode derived echocardiography were performed. Among parameters taken included left ventricular wall dimensions (posterior wall thickness and interventricular septal thickness in diastole), left ventricular end diastolic and systolic dimensions, right ventricular dimension, aortic root dimension and aortic cusp separation. The following indices were derived: Ejection fraction, fractional shortening, and left ventricular mass/index.

Four hundred and fourteen consecutive hypertensive subjects who had echocardiography done and with complete data were recruited. The left ventricular mass index was calculated as

LVM= 0.81 1.04 (interventricular septal thickness + posterior wall thickness + LV end diastolic internal dimension) 3 - (LV end diastolic internal dimension)^3^ +0.6 13

Left atrial dimension was obtained in the M-mode derived echogram in the left parasternal long axis. The maximal left atrial dimension in diastole was taken. An average of three measurements was taken as the mean left atrial dimension. Adebiyi et al. from Ibadan suggested echocardiographic reference in normotensive Nigerians and also noted that sex specific definition of cardiac dimension may not apply to Nigerians.^14^ in this study, left atrial enlargement was defined as LAD >3.7 cm according to the Ibadan echocardiographic reference for normotensive study.^14^

LVM index (LVMI) was calculated as LVM/height in meters2.7. Correcting LVM for height 2.7 minimizes the effect of sex, race, age, and obesity on the validity of various parameters for the diagnosis of left ventricular hypertrophy.^15^,^16^ One adult criterion for LVH is LVMI> 51 g/m2.7.17 As reported by de Simone et al,^17^ adult patients with hypertension and LVMI > 51 g/m2.7 have been found to be at a fourfold greater risk of cardiovascular morbid outcomes. LV geometry was determined after calculation of the relative wall thickness (RWT) by using the formula (2xposterior wall thickness)/LV end diastolic internal dimension.^18^

Left ventricular ejection fraction (EF) was calculated using Teicholz's Formula.^17^ Clinical and demographic parameters including age, sex, weight, height, body mass index, waist circumference, systolic and diastolic blood pressure and pulse pressure were also obtained from their medical records. Participants were consecutive hypertensive subjects who were referred for echocardiography and whose blood pressures were >140/90 mmHg or on antihypertensive therapy.

Data were summarized using means± standard deviation for quantitative variables and frequencies and percentages for quantitative variables. Comparism was done using students' t test and Chi square as appropriate when data was of normal distribution. Statistical analysis was done using the Statistical Package for Social Sciences (SPSS) 16.0. p<0.05 was taken as statistically significant. Institutional Ethical approval was obtained.

## Results

The clinical characteristics of the study participants are shown in table 1. Four hundred and fourteen subjects including 258 males (62.3%) with complete medical and echocardiographic information were subsequently recruited. The mean age was 56.8±16.8 years. Left atrial dilatation (LAD >3.7 cm) was reported in 57.73% of the study participants.

**Table 1:** Patients' characteristics

Variable	
SEX Male(n)	258(62.3%)
Female(n)	156(37.7%)
Age(years)	56.8±16.8
Body mass index(kg/m^2^)	24.2±7.3
Systolic Blood Pressure (mmHg)	142.5±25.2
Diastolic Blood Pressure(mmHg)	84.7±14.6
Waist circumference (cm)	82.5±28.1
Left atrial dilatation (LAD>3.7cm)	239(57.7%)

The clinical and echocardiographic parameters are shown in table 2 between hypertensive subjects with LAD and those with normal left atrial dimension. There was no significant association between sex and left atrial enlargement. Systolic and diastolic blood pressures were similar among hypertensive subjects with left atrial enlargement and those without LAE. The interventricular septal thickness, posterior wall thickness, left ventricular mass and left ventricular mass index were significantly higher among hypertensive subjects with LAE than those without LAE. The frequency of left ventricular hypertrophy was also significantly higher among those with LAE than those without LAE. (65.3% vs. 40.0 % respectively, p<0.05)

**Table 2:** Clinical and echocardiographic parameters between hypertensive subjects with left atrial enlargement and those with normal left atrial dimension

Variable	LAE (239)	Normal LAD (175)	P value
Age	58.2±14.5	54.8 ±19.7	0.043*
Gender M/F	166/92	93/63	0.428
Waist circumference	88.1 ± 26.7	75.8 ±28.4	0.002*
SBP	142.0 ±26.9	143.5 ±21.8	0.695
DBP	85.0 ± 15.6	84.3 ±12.7	0.769
IVSd	13.6 ± 10.1	12.6 ±9.0	0.0308*
PWTd	12.3 ± 3.1	11.5 ± 2.5	0.007*
DT	162.5 ±62.2	165.4 ±68.0	0.662
IVRT	78.0 ±28.3	78.2 ± 22.1	0.978
LVM	290.9 ±35.8	220.3 ±32.1	0.041*
LVMI	79.2 ±12.4	48.7 ±15.5	0.010*
LVH(n)	156 (65.3%)	70 (40.0%)	0.000*

Key to table: SBP-systolic blood pressure, DBP-diastolic blood pressure, IVSd- Interventricular septal thickness in diastole, PWTd- Posterior wall thickness in diastole, DTdeceleration time, IVRT- isovolumic relaxation time, LVM – left ventricular mass, LVMI- left ventricular mass index, LVH-left ventricular hypertrophy.

The association between left ventricular hypertrophy and LAE was studied. The clinical, demographic and echocardiographic characteristics between hypertensive subjects with LVH and those with normal left ventricular mass/index is shown in table 3. LVH in hypertensive subjects is associated with increasing age as the mean age of those with LVH was significantly higher among those with LVH than those without LVH (58.5± 14.4 vs. 53.2 ± 17.3 respectively, p< 0.05). The mean left atrial dimension among those with LVH was significantly hgher than those without LVH (41.4 ±8.4 vs. 35.6 ±5.9 respectively, p<0.05). The left atrial index follows similar pattern. Hypertensive subjects with LVH had a significantly lower ejection fraction, a higher but not statistically significant transmitral E/A velocity than those with normal left ventricular mass/index. The frequency of left ventricular hypertrophy in this study was 54.10%.

**Table 3:** Clinical and echocardiographic characteristics between hypertensives with and without LVH

VARIABLES	LVH(224)	NO LVH(190)	P VALUE
AGE	58.5± 14.4	53.2 ± 17.3	0.023*
GENDER (M/F)	140/84	113/77	0.004*
WAIST	84.9±27.3	78.1±28.8	0.136
SBP	144.5±24.9	138.1 ±18.7	0.360
DBP	85.3±13.5	81.8±13.2	0.236
EF	53.7 ±15.2	62.9 ± 9.6	0.000*
AOD	31.6 ±3.9	30.0 ±4.6	0.112
LAD	41.4 ±8.4	35.6 ±5.9	0.000*
ME/MA	1.0 ±0.6	1.0 ±0.3	0.404
LAD/AOD	1.3 ±0.3	1.3 ±0.8	0.213
LAI	16.0 ±7.2	12.7 ±2.4	0.000*

Key to table: SBP- systolic blood pressure, DBP-diastolic blood pressure, EF- ejection fraction, AOD- aortic root dimension, LAD- left atrial dimension, ME- early mitral inflow velocity, MA- late mitral inflow velocity, LAI- left atrial index.

## Discussion

This study shows that left atrial enlargement is fairly common among treated hypertensive subjects in Nigeria. It also shows that left atrial abnormalities in treated hypertensive subjects are closely related to many clinical and echocardiographic variables. Left atrial enlargement is associated with increasing age and waist circumference in the present study. These are known conventional cardiovascular risk factors with potential for a geometric increase in cardiovascular risk when present. Echocardiographic variables associated with increased left atrial dimension in this study were left ventricular posterior and interventricular septal wall thickness, left ventricular mass and left ventricular mass index.

Evidence suggests that increasing left atrial dimension is associated with higher cardiovascular risk.^18^–^20^ The left atrium performs three functions. First a conduit pipe for transfer of blood from the pulmonary veins to the left ventricle, secondly as a reservoir for blood during the active phase of ventricular contraction and thirdly a contractile function during late atrial systole contributing about 20–30% of the total cardiac output. Therefore, the left ventricular function is closely related to left atrial function both in diastole and in systole.

Left atrial dimension has also been shown to predict the all cause and cardiovascular mortality in the general population.^21^–^23^ In the Losartan Intervention for Endpoint Reduction in Hypertension (LIFE) study, left atrial dimension predicted the all-cause mortality independent of other cardiovascular risk factors.^21^ Other studies have shown that the relationship between left atrial dimension and mortality is dependent on left ventricular mass, left ventricular hypertrophy, left ventricular diastolic dysfunction.^22^–^23^

In this study, the mean left ventricular mass/index and the frequency of left ventricular hypertrophy were significantly higher among hypertensive subjects with left atrial enlargement than those without LAE. The wall dimensions and the other left ventricular parameters which are the major determinants of left ventricular diastolic function were significantly more associated with LAE in this study. This was even recently shown in a group of offspring of hypertensive subjects aged 15–25 years in Ilorin, Nigeria.^24^ Also, among those with left ventricular hypertrophy, mean left atrial dimension and left atrial indices were far significantly higher than those without left ventricular hypertrophy. This agrees with other authors who have shown significant association between left atrial enlargment/abnormalities and left ventricular diastolic function parameters.

Adebiyi et al. in Ibadan in Nigeria showed that the prevalence of LAE in a group of hypertensive subjects was 15.8% using a sex specific classification of LAD>4.2 cm in men or LAD>3.8 in women. They also showed that left atrial size in Nigerian hypertensive subjects is influenced by age, sex, left ventricular mass and left ventricular mass index.^25^ These associations are similar to what we found out in our study except that there was no sex association. This could be due to the fact that we did not use a sex specific definition for left atrial enlargement and the present definition for LAD was derived among normotensive Nigerians. This prevalence is lower to that in this study due to the different definition for LAE in this study. However in another study among normotensive parameters to define normal echocardiographic parameters among Nigerians, the same set of authors suggested that sex specific definition of cardiac dimension may not apply to Nigerians.^14^ in this study, left atrial enlargement was defined as LAD >3.7 cm. Ogah et al showed that left atrial dimension is a strong determinant of left ventricular mass among hypertensive Nigerians.^26^

The prevalence of LAE in this study is also significantly higher among studies from Caucasians. Cuspidi et al. reported a prevalence of 24.5% in women and 21.5% in men using sex specific definitions for LAE.^27^ In a group of 15 pooled study by Cuspidi et al., the prevalence of LAE was found to be between 16–83% using four different criteria for definition of LAE.^28^ They also showed that that there is no significant relationship between sex and LAE among the pooled hypertensive subjects.

LAE is associated with increased prevalence of atrial fibrillation, stroke and cardiovascular mortality.^20^,^25^,^28^ Left atrial volume and left atrial volume index has been shown to be better parameters to detect left atrial enlargement as the M-mode derived echocardiography may overestimate left atrial dimension. However, this was not routinely done in this study and it is a limitation of this study.^29^ The determinants of LAE include age, body mass index, waist circumference, left ventricular mass/index and to a smaller extent systolic blood pressure. 28–29 Most of these parameters were significantly different in the present study between those with LAE and those without LAE except blood pressure, the effect of which has been suggested to be actually related to the clustering of other cardiovascular risk factors.^26^–^27^

## Conclusion

This study shows that using the traditional definition for LAE obtainable from local population, left atrial enlargement is very common among hypertensive subjects in our environment and it is well related to left ventricular hypertrophy, waist circumference, age and left ventricular wall dimensions. Early identification may allow introduction of management options to reduce the prevalence among hypertensive Nigerian subjects. Further studies warranted to determine the clinical profile and consequence of left atrial enlargement among hypertensive Nigerians and to determine their clinical and pathologic significance.

## References

[1] Lang RM, Bierig M, Devereux RB, Flachskampf FA, Foster E, Pellikka PA (2005). Recommendations for chamber quantification: a report from the American Society of Echocardiography's Guidelines and Standards Committee and the Chamber Quantification Writing Group, developed in conjunction with the European Association of Echocardiography, a branch of the European Society of Cardiology. Am J SocEchocardiogr.

[2] Benjamin EJ, D'Agostino RB, Belanger AJ, Wolf PA, Levy D (1995). Left atrial size and the risk of stroke and death. The Framingham Heart Study. Circulation.

[3] Di Tullio MR, Sacco RL, Sciacca RR, Homma S (1999). Left atrial size and the risk of ischemic stroke in an ethnically mixed population. Stroke.

[4] Tani T, Tanabe K, Ono M, Yamaguchi K, Okada M, Sumida T (2004). Left atrial volume and the risk of paroxysmal atrial fibrillation in patients with hypertrophic cardiomyopathy. J Am SocEchocardiogr.

[5] Khurram IM, Dewire J, Mager M, Maqbool F, Zimmerman SL, Zipunnikov V (2013). relationship between left atril appendage morphology and stroke in patients with atrial fibrillation. Heart Rhythm.

[6] Tedesco MA, Di Salvo G, Ratti G, Natale F, Iarussi D, Iacono A (2001). Left atrial size in 164 hypertensive patients: an echocardiographic and ambulatory blood pressure study. ClinCardiol.

[7] Gerdts E, Oikarinen L, Palmieri V, Otterstad JE, Wachtell K, Boman K (2002). Losartan Intervention For Endpoint Reduction in Hypertension (LIFE) Study. Correlates of left atrial size in hypertensive patients with left ventricular hypertrophy: the Losartan Intervention For Endpoint Reduction in Hypertension (LIFE) Study. Hypertension.

[8] Patel DA, Lavie CJ, Milani RV, Shah S, Gilliland Y (2009). Clinical implications of left atrial enlargement: A review. The Ochsner Journal.

[9] Akintunde AA, Familoni OB, Akinwusi PO, Opadijo OG (2010). Relationship between left ventricular geometric pattern on systolic and diastolic function in treated Nigerian hypertensives. Cardiovasc J Afr.

[10] Akintunde AA, Akinwusi PO, Opadijo OG (2010). Left Ventricular hypertrophy, geometric pattern and clinical correlates among treated hypertensive Nigerians. The Pan Afr Med J.

[11] Akintunde AA, Akinwusi PO, Opadijo OG (2010). Left Ventricular hypertrophy, geometric pattern and clinical correlates among treated hypertensive Nigerians. The Pan African Medical Journal.

[12] Sahn DJ, DeMaria A, Kisslo J, Weyman A (1978). Recommendations regarding quantitation in M-mode echocardiography: results of a survey of echocardiography measurements. Circulation.

[13] Devereux RB, Alonso DR, Lutas EM, Gottlieb GJ, Campo E, Sachs I (1986). Echocardiographic assessment of left ventricular hypertrophy: comparison to necropsy findings. Am J Cardiol.

[14] Adebiyi AA, Ogah OS, Aje AA, Ojji DB, Oladapo OO, Falase AO (2006). Echocardiographic reference values in healthy Nigerians. Nigerian Journal of Cardiology.

[15] de Simone G, Daniels SR, Devereux RB, Meyer RA, Roman MJ, de Divitilis O (l992). Left ventricular mass and body size in normotensive children and adults: assessment of allometric relations and impact of overweight. J Am CollCardiol.

[16] Daniels SR, Kimball TR, Morrison JA, Khoury P, Meyer RA (1995). Indexing left ventricular mass to account for differences in body size in children and adolescents without cardiovascular disease. Am J Cardiol.

[17] de Simone G, Devereux RB, Daniels SR, Koren MJ, Meyer RA, Laragh JH (1995). Effect of growth on variability of left ventricular mass: assessment of allometric signals in adults and children and their capacity to predict cardiovascular risk. J Am CollCardiol.

[18] Savage DD, Garrison RJ, Kannel WB, Levy D, Anderson SJ, Stokes J (1987). The spectrum of left ventricular hypertrophy in a general population sample: the Framingham Study. Circulation.

[19] Benjamin EJ, D'Agostino RB, Belanger AJ, Wolfe PA, Levy D (1995). Left atrial size and the risk of stroke and death. The Framingham Heart Study. Circulation.

[20] Di Tullio MR, Sacco RL, Sciacca RR, Homma S (1999). Left atrial size and the risk of ischemic stroke in an ethnically mixed population. Stroke.

[21] Gerdts E, Wachtell K, Omvik P, Otterstad JE, Oikarinen L, Boma K (2007). Left atrial size and risk of major cardiovascular events during antihypertensive treatment: losartan intervention for endpoint reduction in hypertension trial. Hypertension.

[22] Pritchett AM, Maoney DW, Jacobsen SJ, Rodeheffer RJ, Karon BL, Redfield MM (2005). Diastolic dysfunction and left atrial volume: a population-based study. J Am CollCardiol.

[23] Laukkanen JA, Kurl S, Eranen J, Huttunen M, Salonen JT (2005). Left atrium size and the risk of cardiovascular death in middle-aged men. Arch Intern Med.

[24] Kolo PM, Sanya EO, Omotoso AB, Soladoye A, Ogunmodede JA (2012). Left ventricular hypertrophy is associated with diastolic filling alterations in normotensive offspring of hypertensive Nigerians. ISRN Cardiol.

[25] Adebiyi AA, Aje A, Ogah OS, Ojji DB, Dada A, Oladapo OO (2005). Correlates of left atrial size in Nigerian hypertensives. Cardiovasc J Afr.

[26] Ogah OS, Bamgboye AE (2010). Correlates of left ventricular mass in hypertensive Nigerians: an echocardiographic study. Cardiovasc J Afr.

[27] Cuspidi C, Meani S, Fusi V, Valerio C, Catini E, Sala C (2005). Prevalence and correlates of left atrial enlargement in essential hypertension: role of ventricular geometry and the metabolic syndrome: the evaluation of Target organ damage in hypertension study. J Hypertens.

[28] Cuspidi C, Rescoladani M, Sala C (2013). Prevalence of echocardiographic left atrial enlargement in hypertension: A systematic review of recent clinical studies. Am J Hypertens.

[29] Milan A, Puglisi E, Magnino C, Naso D, Abram S, Avenatti E (2012). Left atrial enlargement in essential hypertension: role in the assessment of subclinical hypertensive heart disease. Blood Press.

